# Correlation Between Dietary Index for Gut Microbiota With All‐Cause and Cardiovascular Mortality in Cardiovascular‐Kidney‐Metabolic Syndrome Patients

**DOI:** 10.1002/fsn3.71963

**Published:** 2026-05-28

**Authors:** Anwu Huang, Lianglei Hou, Shanjiang Chen, Bin Lin, Qinqin Zhang

**Affiliations:** ^1^ Department of Cardiology Wenzhou Central Hospital Zhejiang Province China

**Keywords:** all‐cause mortality, cardiovascular‐kidney‐metabolic syndrome, cardiovascular mortality, gut microbiota dietary index

## Abstract

The link between the dietary index for gut microbiota (DI‐GM) and the risk of death in the Cardiovascular‐Kidney‐Metabolic Syndrome (CKM) population remains unclear. Our research investigates the relationship between DI‐GM and mortality in CKM patients. Data from 13,562 individuals diagnosed with CKM, obtained from the National Health and Nutrition Examination Survey (NHANES) spanning 2007 to 2018, were analyzed. Kaplan–Meier curves, Cox regression, smooth curve fitting, and Subgroup analyses were applied to assess the link between DI‐GM and mortality. During the average 82‐months follow‐up period, a total of 1236 deaths occurred, including 371 cases of cardiovascular death. Kaplan–Meier analysis showed that the lowest DI‐GM group exhibited the highest rates of mortality. Multivariable Cox regression found that a one‐point increase in the DI‐GM score was associated with a 10% reduction in all‐cause mortality risk and a 12% decrease in cardiovascular mortality risk. Smooth curve fitting and continuous dose–response estimates indicated a negative linear relationship between DI‐GM scores and both all‐cause and cardiovascular mortality, consistent across various subgroups. This research reveals that higher DI‐GM concentrations in CKM syndrome patients correlate strongly with improved survival outcomes, especially for all‐cause mortality and cardiovascular death among patients in CKM stages 0–3, while also showing significant benefits for all‐cause mortality among patients in CKM stage 4.

## Graphical abstract

This study first demonstrates that in CKM syndrome patients, a higher dietary index for gut microbiota (DI‐GM) is associated with lower all‐cause and cardiovascular mortality. Each 1‐point increase in DI‐GM reduces all‐cause mortality risk by 10% and cardiovascular mortality by 12%. The inverse association is especially significant in CKM stages 0–3, suggesting that gut‐microbiota‐targeted dietary optimization may improve prognosis.

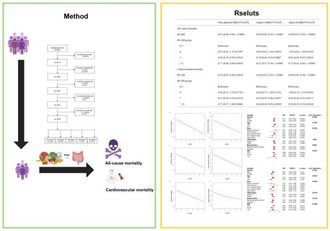

## 1 Introduction

CKM was officially proposed by the American Heart Association in 2023 as a new clinical concept, it demonstrates the intricate relationship between metabolic disorders, kidney impairment, and heart conditions (Ndumele, Rangaswami, et al. 2023). These intertwined pathologies significantly exacerbate the adverse outcomes of cardiovascular disease (CVD) in affected individuals (Marassi and Fadini 2023). Research conducted between 2015 and 2020 revealed that one in four American adults were diagnosed with CKM syndrome, with associated healthcare expenditures consuming over three‐quarters of the nation's total medical spending (Ostrominski et al. 2023). To facilitate management, the AHA has categorized CKM into five stages, reflecting its progressive nature, with stages CKM0‐3 representing the preclinical phase (Ndumele, Neeland, et al. 2023).

In recent years, the influence of the gut microbiota (GM) in metabolic disorders, CVDs, and chronic kidney disease (CKD) has garnered significant research attention. Dysbiosis of the GM can lead to chronic inflammation, impaired insulin response, and endothelial dysfunction—key characteristics of CKM syndrome (Jonsson and Bäckhed 2017; Khuu et al. 2025). Diet plays a critical function in shaping gut microbial diversity and balance. High‐fat diets can disrupt intestinal integrity, deplete beneficial bacteria, and promote the overgrowth of pathogenic microbes, triggering a cascade of negative health effects, including metabolic dysfunction and cardiovascular stress (Fu et al. 2025; Jia et al. 2023; Ross et al. 2024; Van Hul and Cani 2023). However, diets abundant in dietary fiber work wonders for gut microbiota composition and diversity, boost short‐chain fatty acid production, enhance insulin sensitivity, and cut down the developing CKM syndrome (Musolino et al. 2025; Pushpass et al. 2022). DI‐GM, developed by Kase (Kase et al. 2024), quantifies how daily dietary choices affect gut microbiome health. The DI‐GM index encompasses 14 nutritional factors, with 10 being advantageous and 4 posing risks. The higher the DI‐GM score is, the healthier the diet is and the more beneficial it is to the health of the intestinal microbiota.

Prior research indicates a correlation between DIGM and mortality of patients with diabetes or prediabetes (Song et al. 2025), patients with fatty liver disease related to metabolic dysfunction (Wu et al. 2025), and patients with metabolic syndrome (Zheng et al. 2025). However, most of these studies have concentrated on single disease entities, leaving a significant knowledge void regarding DI‐GM's function within the broader context of CKM syndrome. CKM syndrome is marked by a triad of intertwined issues involving metabolic, renal, and cardiovascular malfunctions. Notably, the AHA's CKM staging framework offers a unique opportunity to move beyond disease‐specific analyses, capturing the cumulative burden and temporal evolution of multisystem dysregulation. Unlike traditional binary classifications of metabolic or CVDs, CKM staging integrates preclinical risk factors (stages 0–3) and clinically established disease (stage 4), allowing for a more nuanced exploration of how diet—and specifically DI‐GM‐directed nutrition—may vary across disease severity. This approach may uncover stage‐dependent opportunities for dietary intervention that are often overlooked when considering metabolically burdened populations as a homogeneous group. For example, the impact of DI‐GM‐mediated pathways on mortality could differ between early metabolic dysregulation and advanced CVD, informing targeted nutritional strategies.

Thus, this study investigated the connection between DI‐GM and both all‐cause and cardiovascular mortality in CKM syndrome individuals, with a particular focus on how CKM stage modifies these associations. By leveraging the staged classification of CKM, we seek to determine whether a DI‐GM‐favorable diet is consistent across the CKM severity or whether it is primarily driven by associations in preclinical stages.

## 2 Method

This longitudinal cohort study utilized data from the NHANES, a continuous research initiative led by the CDC's National Center for Health Statistics (NCHS). NHANES uses a complex, stratified, multistage probability sampling design to assess the health status of U.S. civilians. The study integrates detailed interviews, physiological evaluations, and laboratory tests to gather holistic information about participant demographics, health statuses, and dietary habits. All procedures followed in NHANES received formal approval from the NCHS Ethics Committee, with written informed consent obtained from every participant. Since the analysis utilized anonymized, publicly accessible data, no further approval by an institutional ethics committee was required.

**FIGURE 1:**
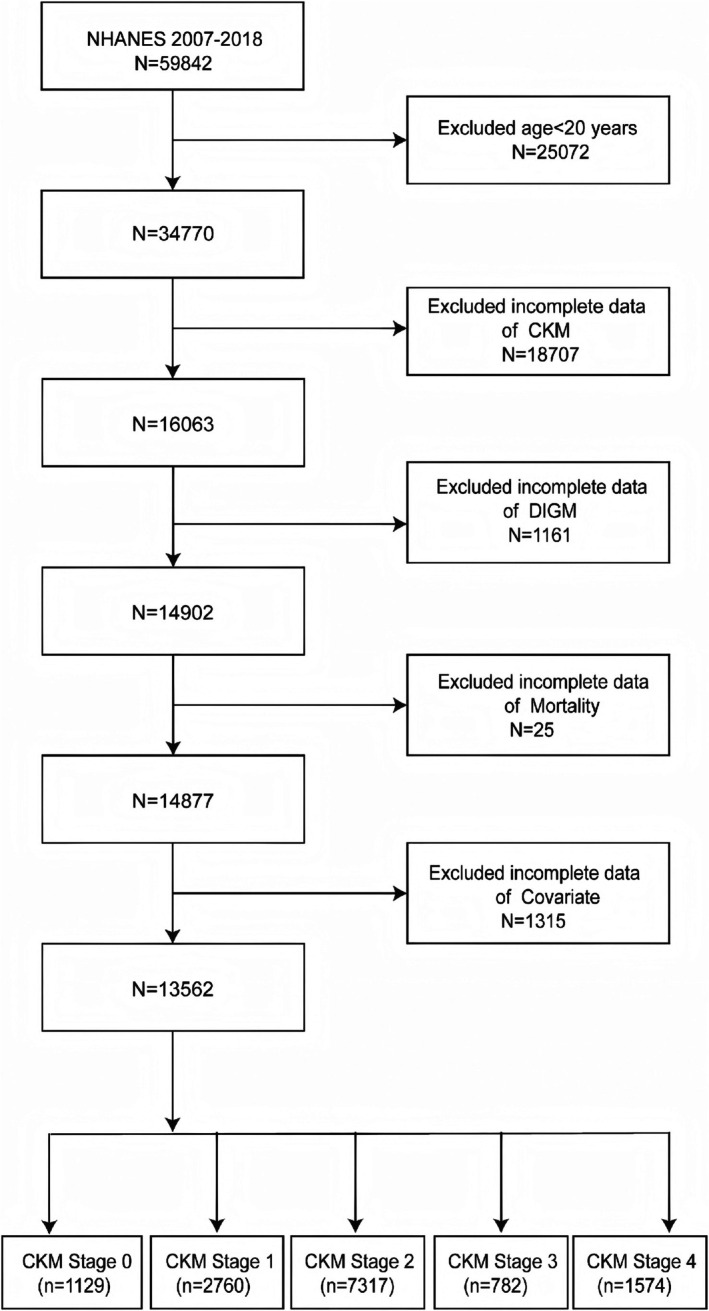
Selection flowchart of the research population.

This study utilized NHANES data spanning 2007–2018. The study began with a pool of 59,842 participants. Following the established eligibility criteria, individuals under 20 years of age (*N* = 25,072) were excluded. Further exclusions were made for missing or incomplete data on CKM (*N* = 18,707), DI‐GM (*N* = 1161), mortality outcomes (*N* = 25), and other covariates (*N* = 1315). After this screening process, 13,562 participants remained for analysis. The distribution of participants across CKM stages 0–4 was as follows: 1129 in CKM0, 2760 in CKM1, 7317 in CKM2, 782 in CKM3, and 1574 in CKM4. Figure 1 illustrates the participant selection process in detail.

### 2.1 Definition of DI‐GM

DI‐GM is a rating instrument crafted by Kase et al. (Kase et al. 2024) for evaluating the influence of daily diet on the health of the intestinal microbiome. In simple terms, the healthier the diet, the better it is for the health of the gut microbiota, and the higher the DI‐GM score will be. The DI‐GM score incorporates 14 dietary components. Beneficial factors include fermented dairy products, soybeans, chickpeas, avocados, broccoli, coffee, fiber, cranberries, whole grains, and tea, while harmful components consist of unprocessed and processed red meats, refined cereals, and high‐fat diets. The DI‐GM scoring utilized data from two separate 24‐h dietary recall surveys conducted by NHANES. Initial data collection took place in person, followed by a follow‐up interview via telephone within 3 to 10 days. Inconsistent energy intake was excluded using the food and nutrition database from the dietary study, and the average nutrient intake over these two days was calculated. Advantageous components earn one point when intake meets or surpasses the median for one's gender group. For the unfavorable factors, 1 point is awarded if consumption falls below the same‐sex median or a diet with a high‐fat content accounting for less than 40%. The total score range of DIGM is from 0 to 14 points. Table S1 provides the full scoring guidelines for DI‐GM. Previous studies indicated DI‐GM score of 0–3 correlate strongly with poor clinical prognoses, while a score of ≥ 6 is considered to be of low risk (Liu and Huang 2025; Zhang et al. 2025). Therefore, in this study, we divided the scores of DI‐GM into four groups according to previous research:0–3, 4, 5, ≥ 6. We conducted additional exploratory analyses, dividing the range of 0–2, 3, 4, 5, 6, 7, 8–12 into 7 groups, and obtained the continuous dose–response estimates.

### 2.2 Definition of CKM Syndrome

CKM syndrome stages are classified 0–4 according to AHA standards (Ndumele, Neeland, et al. 2023). The classification system is as follows: Stage 0 encompasses individuals who have a standard body mass index, waist measurement, normal blood sugar levels, regular blood pressure, healthy cholesterol levels, and no signs of chronic kidney disease or any form of cardiovascular illness, whether it's mild or severe. Stage 1 includes those with obesity, abdominal obesity, or impaired fat tissue, absent other metabolic risks or chronic kidney disease. Stage 2 marks metabolic complications beginning to surface, encompassing elevated triglyceride levels, high blood pressure, diabetes mellitus, metabolic syndrome, and chronic kidney disease classified as moderate‐to‐high risk according to KDIGO. Stage 3 indicates early, non‐symptomatic cardiovascular complications, with no clinical CVD but the presence of at least two criteria: KDIGO‐defined very high‐risk CKD or 10‐year CVD risk ≥ 20%. Additionally, any of the following criteria may be present: excess weight, central adiposity, pre‐diabetic state, elevated triglycerides, high blood pressure, diabetic condition, metabolic syndrome, or significant kidney impairment. cardiovascular disease alongside additional metabolic complications such as being overweight or obese, particularly with excess abdominal fat, prediabetic conditions, elevated triglyceride levels, high blood pressure, diabetes, metabolic syndrome, or chronic kidney disease classified as moderate to high or even very high risk based on KDIGO guidelines. A more comprehensive description of the CKM syndrome staging can be found in Table S2.

### 2.3 Definition of Mortality

The primary outcomes of this study were all‐cause and cardiovascular mortality. Mortality status and causes were determined using the 2019 NHANES Public‐use Linked Mortality File, which tracks mortality data for NHANES participants connected to NCHS up to December 31, 2019. Duration of follow‐up for each subject was measured from the survey examination date to the earliest of their death or the conclusion of the follow‐up period.

### 2.4 Definition of Covariates

We collected a comprehensive set of covariates from the NHANES database, chosen for known or possible links to mortality. Information on age, race, gender, marital status, and smoking status was gathered via self‐report during in‐home interviews. BMI was determined by dividing the individual's weight in kilograms by the square of their height in meters. Hypertension was characterized as systolic pressure ≥ 140 mmHg or diastolic pressure ≥ 90 mmHg, a doctor's diagnosis, or current antihypertensive drug use. Similarly, diabetes was identified through a doctor's report, a fasting blood sugar level of 126 mg/dL or higher, a HbA1c level of 6.5% or more, or the reliance on insulin or oral diabetes drugs. Standardized testing methods were employed for biochemical analyses, and the following biochemical parameters were included as covariates: aspartate aminotransferase (AST), uric acid, alanine aminotransferase (ALT), blood urea nitrogen (BUN), creatinine, high‐density lipoprotein (HDL) cholesterol, total cholesterol (TC), low‐density lipoprotein (LDL) cholesterol, and triglycerides (TG).

### 2.5 Statistical Analysis

Baseline characteristics are presented in the form of mean ± standard deviation, median ± interquartile range, and in the form of frequency (percentage). For continuous variables, ANOVA and Kruskal‐Wallis tests were applied based on the normality of the data. Categorical variables were analyzed using Chi‐square or Fisher's exact tests, depending on the specific circumstances. A Kaplan–Meier curve was drawn to visually present mortality probabilities among the different DI‐GM groups during the follow‐up period. Cox regression analysis evaluated the link between DI‐GM and mortality, guided by a directed acyclic graph (Figure S1). Three models were constructed: the non‐adjusted model, Adjust I model (adjusted for age, gender, race, marital status, and smoking status, based on the DAG), and Adjust II model (adjusted for age, gender, race, marital status, ALT, urea nitrogen, uric acid, TC, AST, creatinine, TG, HDL, LDL, BMI, diabetes, hypertension, and smoking status, accounting for both confounding and mediating factors, based on the DAG). Schoenfeld residuals confirmed the Cox regression models met the proportional hazards (PH) assumption. To assess the relationship between continuous DI‐GM and mortality, smooth curve fitting and continuous dose–response analyses were performed. Finally, subgroup analyses assessed the DI‐GM–mortality link for robustness. Statistical analyses were conducted with R and EmpowerStats, with significance set at *p* < 0.05.

## 3 Results

### 3.1 Baseline Characteristics

A total of 13,562 participants were included in the final analysis. Based on the DI‐GM score, participants were categorized into four groups: 0–3 (*N* = 2526), 4 (*N* = 2926), 5 (*N* = 3112), and ≥ 6 (*N* = 4998). The baseline characteristics of all participants, stratified by DI‐GM groups, are summarized in Table 1. Notable variations in the demographic profiles of the groups were detected. Higher DI‐GM scorers were significantly older (*p* < 0.001) and a greater female representation (*p* < 0.001). Statistically significant differences were also noted in race and marital status distributions (both *p* < 0.001). Regarding laboratory parameters, higher DI‐GM groups showed lower levels of AST, urea nitrogen, creatinine, uric acid, and BMI compared to lower DI‐GM groups (all *p* < 0.01). TC and HDL levels were elevated in higher group (*p* = 0.006 and *p* < 0.001, respectively), while no notable variations were observed in ALT, triglyceride, or LDL levels between groups (all *p* > 0.05). Individuals exhibiting greater DI‐GM values demonstrated a markedly reduced likelihood of diabetes (*p* = 0.001) alongside a significantly larger segment of non‐smokers (*p* < 0.001). Nevertheless, the groups showed no statistically meaningful distinction when it came to hypertension rates (*p* = 0.242). A notable variation in CKM stage distribution was found among the DI‐GM score categories (*p* < 0.001).

**TABLE 1:** Baseline characteristics of participants.

Characteristics	DI‐GM				*p*
	0–3	4	5	≥ 6	
*N*	2526	2926	3112	4998	
Age (years)	48.87 ± 17.78	48.66 ± 17.79	49.55 ± 17.47	51.45 ± 17.38	< 0.001
Gender (*n*, %)					< 0.001
Male	1322 (52.34%)	1488 (50.85%)	1490 (47.88%)	2230 (44.62%)	
Female	1204 (47.66%)	1438 (49.15%)	1622 (52.12%)	2768 (55.38%)	
Race (*n*, %)					< 0.001
Mexican American	394 (15.60%)	492 (16.81%)	547 (17.58%)	646 (12.93%)	
Other Hispanic	270 (10.69%)	336 (11.48%)	361 (11.60%)	514 (10.28%)	
Non‐Hispanic White	959 (37.97%)	1151 (39.34%)	1274 (40.94%)	2359 (47.20%)	
Non‐Hispanic Black	683 (27.04%)	670 (22.90%)	613 (19.70%)	766 (15.33%)	
Other Races	220 (8.71%)	277 (9.47%)	317 (10.19%)	713 (14.27%)	
Marital status (*n*, %)					< 0.001
Married or living with partner	1465 (58.00%)	1644 (56.19%)	1901 (61.09%)	3140 (62.83%)	
Never married and others	1061 (42.00%)	1282 (43.81%)	1211 (38.91%)	1858 (37.17%)	
ALT (U/L)	25.28 ± 19.54	25.35 ± 24.36	24.81 ± 18.69	24.25 ± 16.13	0.413
AST (U/L)	25.29 ± 22.95	25.58 ± 24.29	25.20 ± 19.40	24.99 ± 17.08	< 0.001
Urea nitrogen (mg/dL)	14.20 ± 6.89	13.78 ± 6.20	13.46 ± 5.80	13.51 ± 5.48	0.004
Total cholesterol (mg/dL)	189.51 ± 41.71	190.82 ± 40.96	192.45 ± 42.04	192.32 ± 40.55	0.006
Creatinine (mg/dL)	0.93 ± 0.61	0.91 ± 0.57	0.88 ± 0.39	0.87 ± 0.39	< 0.001
Triglyceride (mg/dL)	120.06 ± 67.29	122.79 ± 69.59	122.95 ± 70.15	119.56 ± 67.39	0.130
Uric acid (mg/dL)	5.60 ± 1.47	5.53 ± 1.48	5.48 ± 1.43	5.41 ± 1.38	< 0.001
HDL (mg/dL)	53.02 ± 15.64	53.34 ± 16.01	53.86 ± 15.51	55.40 ± 16.27	< 0.001
LDL (mg/dL)	113.03 ± 36.49	113.28 ± 35.38	114.22 ± 36.25	113.13 ± 34.84	0.428
BMI, kg/m^2^	30.00 ± 7.35	29.34 ± 7.01	29.23 ± 6.99	28.64 ± 6.60	< 0.001
Diabetes (*n*, %)					0.001
Yes	381 (15.08%)	399 (13.64%)	403 (12.95%)	593 (11.86%)	
No	2145 (84.92%)	2527 (86.36%)	2709 (87.05%)	4405 (88.14%)	
Hypertension (*n*, %)					0.242
Yes	1554 (61.52%)	1851 (63.26%)	1995 (64.11%)	3165 (63.33%)	
No	972 (38.48%)	1075 (36.74%)	1117 (35.89%)	1833 (36.67%)	
Smoke status (*n*, %)					< 0.001
0	1296 (51.31%)	1564 (53.45%)	1731 (55.62%)	2962 (59.26%)	
1	1230 (48.69%)	1362 (46.55%)	1381 (44.38%)	2036 (40.74%)	
CKM stages (*n*, %)					< 0.001
0	170 (6.73%)	237 (8.10%)	255 (8.19%)	467 (9.34%)	
1	503 (19.91%)	552 (18.87%)	634 (20.37%)	1071 (21.43%)	
2	1352 (53.52%)	1605 (54.85%)	1709 (54.92%)	2651 (53.04%)	
3	155 (6.14%)	161 (5.50%)	170 (5.46%)	296 (5.92%)	
4	346 (13.70%)	371 (12.68%)	344 (11.05%)	513 (10.26%)	

Abbreviations: ALT, Alanine Aminotransferase; AST, Aspartate Aminotransferase; BMI, Body mass index; CKM, Cardiovascular‐kidney‐metabolicl; HDL, High density lipoprotein cholesterol; LDL, Low density lipoprotein cholesterol.

### 3.2 Kaplan–Meier Analysis Between DI‐GM Score and Mortality in the CKM Population

**FIGURE 2:**
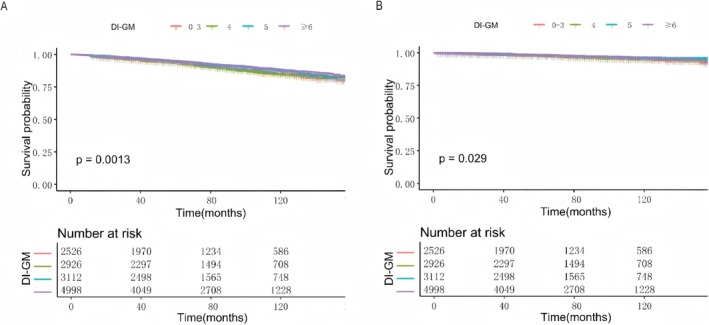
Kaplan–Meier curves analysis of the relationship between DI‐GM and mortality in adults with CKM syndrome. (A) DI‐GM and all‐cause mortality. (B) DI‐GM and cardiovascular mortality.

During the CKM cohort follow‐up, mortality outcomes were assessed across DI‐GM score categories using Kaplan–Meier analysis (Figure 2). The log‐rank test showed significant differences in all‐cause mortality between DI‐GM groups (*p* = 0.0013, Figure 2A). The survival curves indicate that elevated DI‐GM scores correlate with greater cumulative survival rates. Similarly, DI‐GM scores were significantly correlated with cardiovascular mortality (*p* = 0.029, Figure 2B).

### 3.3 Association Between DI‐GM Score and Mortality in the CKM Population

Table 2 shows that in multivariate Cox regression, the DI‐GM score independently reduced risks of all‐cause and cardiovascular mortality. After accounting for all potential confounding factors, every incremental rise in the DI‐GM score correlated with a 10% reduction in overall mortality risk (hazard ratio 0.90, *p* < 0.0001) and a more pronounced 12% drop in cardiovascular‐related deaths (hazard ratio 0.88, *p* < 0.0001). Upon categorizing the DI‐GM scores, the data revealed a striking trend: individuals in the top tier exhibited a 28% reduction in all‐cause mortality risk (hazard ratio = 0.72, *p* < 0.0001) and a 30% decrease in cardiovascular‐related deaths (hazard ratio = 0.70, *p* = 0.0148) when stacked against their counterparts in the lowest bracket.

**TABLE 2:** Multivariable Cox regression analysis of the relationship between DI‐GM. and mortality in adults with CKM syndrome.

	Non‐adjusted [HR (95% CI) *p*]	Adjust I [HR (95% CI) *p*]	Adjust II [HR (95% CI) *p*]
All‐cause mortality			
DI‐GM	0.93 (0.90, 0.96) < 0.0001	0.88 (0.86, 0.91) < 0.0001	0.90 (0.87, 0.93) < 0.0001
DI‐GM group			
0–3	Reference	Reference	Reference
4	0.97 (0.82, 1.15) 0.7607	0.98 (0.83, 1.16) 0.8026	1.02 (0.86, 1.20) 0.8549
5	0.83 (0.70, 0.98) 0.0316	0.78 (0.66, 0.93) 0.0047	0.82 (0.69, 0.97) 0.0223
≥ 6	0.77 (0.66, 0.90) 0.0010	0.67 (0.57, 0.78) < 0.0001	0.72 (0.61, 0.84) < 0.0001
Cardiovascular mortality			
DI‐GM	0.91 (0.85, 0.96) 0.0014	0.86 (0.81, 0.92) < 0.0001	0.88 (0.83, 0.94) < 0.0001
DI‐GM group			
0–3	Reference	Reference	Reference
4	0.96 (0.71, 1.29) 0.7726	0.96 (0.71, 1.30) 0.7931	1.00 (0.74, 1.35) 0.9842
5	0.67 (0.49, 0.93) 0.0150	0.62 (0.45, 0.86) 0.0037	0.66 (0.48, 0.91) 0.0113
≥ 6	0.75 (0.57, 1.00) 0.0464	0.64 (0.48, 0.85) 0.0020	0.70 (0.53, 0.93) 0.0148

*Note:* Non‐adjusted model: No adjustments. Adjust I model: Adjusted for age, gender, race, marital status, and smoking status. Adjust II model: Adjusted for age, gender, race, marital status, ALT, AST, urea nitrogen, total cholesterol, creatinine, triglycerides, uric acid, HDL, LDL, BMI, diabetes, hypertension, and smoking status.

Additionally, correlations between DI‐GM scores and all‐cause and cardiovascular mortality were evaluated across CKM syndrome stages (Tables S3 and S4). For individuals classified in CKM stages 0–3 (preclinical phase), every single‐point uptick in DI‐GM corresponded to a 10% reduction in the likelihood of death from any cause (HR = 0.90, 95% CI: 0.87–0.94, *p* < 0.0001) and a 14% decrease in cardiovascular‐related mortality risk (HR = 0.86, 95% CI: 0.79–0.93, *p* = 0.0004) when all variables were factored into the analysis. When stacked against the cohort with the lowest DI‐GM scores (0–3), participants boasting a DI‐GM of 6 or higher enjoyed a 28% lower chance of dying from any cause (HR = 0.72, 95% CI: 0.59–0.88, *p* = 0.0017) and saw their cardiovascular mortality risk shrink by a substantial 39% (HR = 0.61, 95% CI: 0.41–0.91, *p* = 0.0157). In contrast, among patients with CKM stage 4 (established CVD), the protective association was attenuated. Although higher DI‐GM score was still significantly linked to reduced all‐cause mortality (with each one‐point increase corresponding to an HR of 0.92, 95% CI: 0.87–0.97, *p* = 0.0043; and those scoring ≥ 6 versus 0–3 showing an HR of 0.73, 95% CI: 0.56–0.95, *p* = 0.0187), there was no meaningful connection to cardiovascular mortality (each one‐point increase: HR = 0.93, 95% CI: 0.85–1.02, *p* = 0.1492).

### 3.4 Dose–Response Relationship Between DI‐GM Score and Mortality in the CKM Population

**FIGURE 3:**
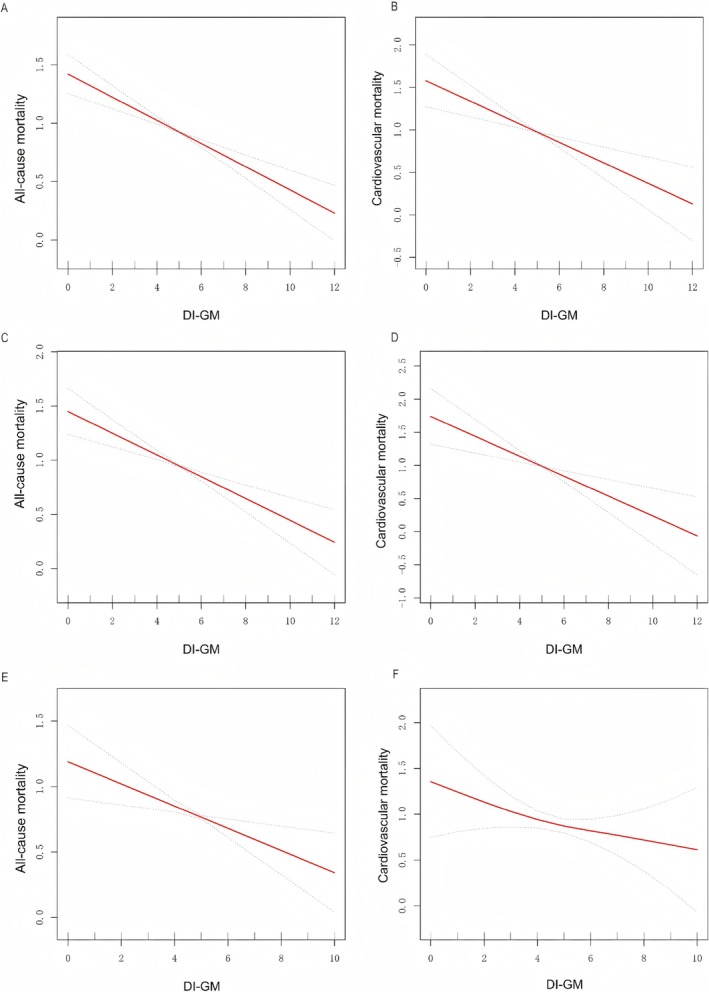
Smooth curve fitting analysis of the relationship between DI‐GM and mortality in adults with CKM syndrome. (A) DI‐GM and all‐cause mortality in CKM stages 0–4. (B) DI‐GM and cardiovascular mortality in CKM stages 0–4. (C) DI‐GM and all‐cause mortality in CKM stages 0–3. (D) DI‐GM and cardiovascular mortality in CKM stages 0–3. (E) DI‐GM and all‐cause mortality in CKM stage 4. (F) DI‐GM and cardiovascular mortality in CKM stage 4. Adjust for: Age, gender, race, marital status, ALT, AST, urea nitrogen, total cholesterol, creatinine, triglycerides, uric acid, HDL, LDL, BMI, diabetes, hypertension, smoking status.

The connection between DI‐GM scores and mortality risk was further examined through smooth curve fitting analysis (Figure 3). The results revealed a clear, dose‐dependent inverse linear relationship, indicating that elevated DI‐GM scores were consistently correlated with lower risks of both all‐cause and cardiovascular mortality. This trend was observed across the total population (Figure 3A,B), the CKM 0–3 stages subgroup (Figure 3C,D), and the CKM 4 stage subgroup (Figure 3E,F). Based on the distribution of the sample size (Table S5), this study combined several smaller groups and re‐categorized DI‐GM into seven groups (0–2, 3, 4, 5, 6, 7, 8–12). Continuous dose–response estimates also demonstrated that as DI‐GM scores increased, all‐cause and cardiovascular mortality decreased across the total population (Table 3).

**TABLE 3:** Continuous dose–response estimates of the relationship between DI‐GM. and mortality in adults with CKM syndrome.

	Non‐adjusted [HR (95% CI) *p*]	Adjust I [HR (95% CI) *p*]	Adjust II [HR (95% CI) *p*]
All‐cause mortality			
DI‐GM group			
0–2	Reference	Reference	Reference
3	0.75 (0.58, 0.96) 0.0237	0.75 (0.58, 0.97) 0.0266	0.75 (0.58, 0.97) 0.0263
4	0.81 (0.64, 1.01) 0.0663	0.81 (0.65, 1.02) 0.0776	0.84 (0.67, 1.06) 0.1466
5	0.69 (0.55, 0.87) 0.0014	0.65 (0.52, 0.82) 0.0002	0.68 (0.54, 0.86) 0.0011
6	0.66 (0.52, 0.84) 0.0008	0.63 (0.50, 0.80) 0.0002	0.67 (0.53, 0.86) 0.0013
7	0.70 (0.54, 0.90) 0.0060	0.56 (0.44, 0.73) < 0.0001	0.60 (0.46, 0.78) 0.0001
≥ 8	0.50 (0.37, 0.68) < 0.0001	0.40 (0.29, 0.54) < 0.0001	0.43 (0.32, 0.59) < 0.0001
Cardiovascular mortality			
DI‐GM group			
0–2	Reference	Reference	Reference
3	0.57 (0.37, 0.88) 0.0117	0.55 (0.36, 0.86) 0.0083	0.57 (0.36, 0.88) 0.0117
4	0.68 (0.46, 0.99) 0.0465	0.67 (0.46, 0.98) 0.0386	0.70 (0.48, 1.03) 0.0735
5	0.48 (0.32, 0.71) 0.0003	0.43 (0.29, 0.64) < 0.0001	0.46 (0.31, 0.69) 0.0002
6	0.59 (0.40, 0.89) 0.0107	0.53 (0.36, 0.80) 0.0022	0.58 (0.39, 0.87) 0.0091
7	0.57 (0.36, 0.88) 0.0120	0.45 (0.29, 0.70) 0.0004	0.50 (0.32, 0.78) 0.0023
≥ 8	0.36 (0.21, 0.63) 0.0003	0.27 (0.16, 0.48) < 0.0001	0.31 (0.18, 0.55) < 0.0001

*Note:* Non‐adjusted model: No adjustments. Adjust I model: Adjusted for age, gender, race, marital status, and smoking status. Adjust II model: Adjusted for age, gender, race, marital status, ALT, AST, urea nitrogen, total cholesterol, creatinine, triglycerides, uric acid, HDL, LDL, BMI, diabetes, hypertension, and smoking status.

### 3.5 Subgroup Analyses Between DI‐GM Score and Mortality in the CKM Population

**FIGURE 4:**
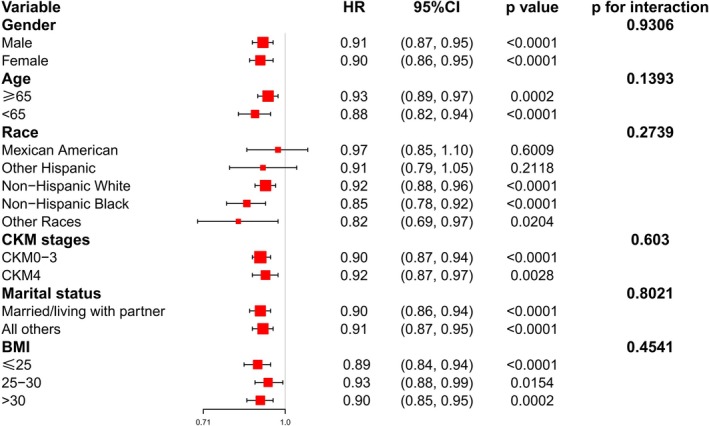
Forest plots of subgroup analyses for the relationship between DI‐GM and all‐cause mortality in adults with CKM syndrome. Adjust for: Age, gender, race, marital status, ALT, AST, urea nitrogen, total cholesterol, creatinine, triglycerides, uric acid, HDL, LDL, BMI, diabetes, hypertension, smoking status, excluding the stratification factor itself.

**FIGURE 5:**
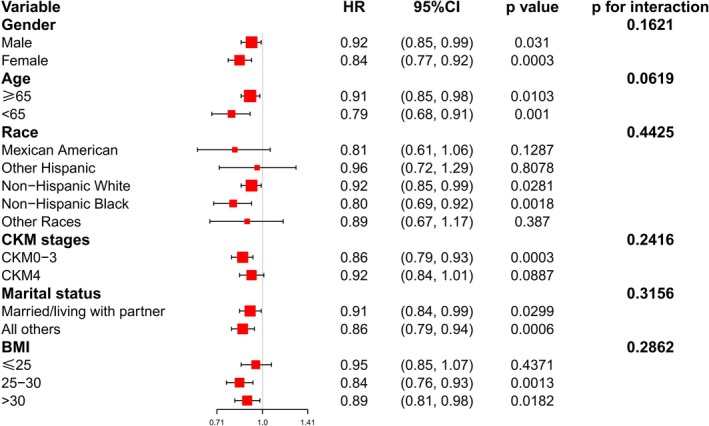
Forest plots of subgroup analyses for the relationship between DI‐GM and cardiovascular mortality in adults with CKM syndrome. Adjust for: Age, gender, race, marital status, ALT, AST, urea nitrogen, total cholesterol, creatinine, triglycerides, uric acid, HDL, LDL, BMI, diabetes, hypertension, smoking status, excluding the stratification factor itself.

The subgroup analyses depicted in Figures 4 and 5 revealed that the inverse relationship between DI‐GM and both all‐cause and cardiovascular mortality held steady across the board for every subgroup we scrutinized. When it came to all‐cause mortality, the interaction *p*‐values came in at 0.9306 for gender, 0.1393 for age, 0.2739 for race, 0.603 for CKM stage, 0.8021 for marital status, and 0.4541 for BMI. As for cardiovascular mortality, the interaction *p*‐values were 0.1621 for gender, 0.0619 for age, 0.4425 for race, 0.2416 for CKM stage, 0.3156 for marital status, and 0.2862 for BMI.

## 4 Discussion

This research initially demonstrates that maintaining a gut‐friendly diet quantified by the DI‐GM score is associated with any cause or cardiovascular‐related death among adults with CKM syndrome. Specifically, a rise of one unit in the DI‐GM index correlated with a 10% decrease in overall mortality rates and a 12% decline in cardiovascular mortality rates after comprehensive adjustment for demographic, clinical, and lifestyle factors. These findings were consistently supported by Kaplan–Meier survival analysis, multivariable cox regression, smooth curve fitting, and subgroup analyses, highlighting the consistent connection across diverse populations. The consistent and graded inverse relationship underscores the importance of dietary quality in the CKM syndrome.

While prior research has established the correlation of the DI‐GM in specific populations, our study extends this evidence to the broader and clinically complex CKM syndrome population. Consistent with earlier studies in patients with MASLD (Wu et al. 2025), MetS (Zheng et al. 2025), and diabetes or prediabetes (Zheng et al. 2025; Wang et al. 2025), elevated DI‐GM scores correlated with lower overall and cardiac‐related death rates. Specifically, this study found that every single point uptick in the DI‐GM score corresponded to a 10% reduction in overall mortality and a 12% decrease in cardiovascular mortality. Importantly, the study further explores this association across the severity spectrum of CKM. The inverse linear association between DI‐GM and cardiovascular mortality was maintained in CKM stages 0–3, but became less pronounced and statistically insignificant in stage 4. Several factors may explain this attenuation. First, in advanced CKM stage 4, the substantial burden of established CVD and competing risks might diminish the relative contribution of dietary factors to cardiovascular outcomes. Second, potential measurement and misclassification errors in dietary assessments, limited statistical power due to small sample sizes, and residual confounding by disease severity, medication use, and unmeasured lifestyle factors may have biased the results toward the null. Further studies with more precise exposure assessments and detailed adjustments for disease severity are necessary to clarify the role of DI‐GM in advanced CKM stages. This stage‐specific analysis adds a new dimension to previous DI‐GM studies focused on single disease entities like MASLD or diabetes, highlighting the complex relationship between nutrition, microbiome balance, and systemic dysfunction across the CKM spectrum.

The link between DI‐GM and reduced mortality in CKM syndrome involves multiple pathways. A greater DI‐GM shows a dietary pattern rich in beneficial components such as dietary fiber, fermented dairy products, and legumes. These foods promote the proliferation of beneficial gut bacteria, enhance the production of short‐chain fatty acids (SCFAs), particularly butyrate, and consequently improve insulin sensitivity, reduce systemic inflammation, and help maintain glycemic stability (Cui et al. 2022; Tolhurst et al. 2012; Valdes et al. 2018). Additionally, the dietary pattern advocated by DI‐GM can suppress the overgrowth of harmful bacteria, reduce the conversion of choline and carnitine to trimethylamine, thereby decreasing liver‐derived trimethylamine‐N‐oxide (TMAO) levels. Elevated TMAO concentrations are closely associated with atherosclerosis, thrombosis, and cardiovascular risk (Li et al. 2022; Witkowski et al. 2020; Zhuang et al. 2019). Furthermore, a favorable GM composition may exert protective effects against diabetes and its cardiovascular complications by modulating immune responses, enhancing gut barrier function, and influencing host metabolic pathways, ultimately contributing to the observed reduction in mortality risk (Han et al. 2025; Koren et al. 2012).

To elucidate the clinical relevance of DI‐GM, a comparison with established dietary quality indices is necessary. Widely used indices such as the Mediterranean Diet Score (MDS) and the Healthy Eating Index (HEI) are primarily based on epidemiological evidence linking dietary patterns to cardiometabolic disease risk. The HEI evaluates adherence to U.S. Dietary Guidelines, emphasizing nutritional adequacy and balance across food groups (Krebs‐Smith et al. 2018). The MDS assesses adherence to the traditional Mediterranean diet, focusing on the intake of olive oil, fish, vegetables, whole grains, and fruits (Trichopoulou et al. 2003). In contrast, DI‐GM is not derived from direct associations with disease endpoints but from research on the relationship between food intake and GM composition. Its components are selected based on microbiome research, aiming to quantify the diet's impact on gut microbial ecosystem health. Therefore, DI‐GM's primary advantage lies in its focus on the specific biological mechanisms underlying the “diet‐microbiome” axis. In clinical practice, DI‐GM can complement traditional dietary indices, especially in contexts where gut microecology plays a key role in health or when precise nutritional interventions targeting the GM are required.

The findings of this study further demonstrate significant clinical relevance. First, DI‐GM serves as a simple and quantifiable dietary index, offering clinicians a practical tool for assessing mortality risk in CKM syndrome patients. Second, as a straightforward dietary pattern score, clinicians can guide patients, particularly those with low DI‐GM scores, to increase their consumption of beneficial foods, such as cultured dairy, legumes, unrefined cereals, and vegetables, while reducing the intake of red meats and processed grains. Finally, the association between improved diet quality and reduced overall mortality persists even in CKM stage 4, suggesting that dietary optimization targeting the gut microbiome should be an integral part of CKM syndrome management across all stages.

To our knowledge, this is the first study to investigate the relationship between DI‐GM and mortality in a dedicated CKM population. Previous research has largely focused on individual dietary components or general dietary patterns in relation to metabolic or cardiovascular outcomes. This study extends these findings by applying DI‐GM to the CKM syndrome patient population and utilizing its staging framework to demonstrate its correlation with mortality rates. This approach highlights the added value of the CKM staging method beyond traditional disease‐specific analyses.

Nonetheless, it is crucial to recognize the various limitations of our investigation. Firstly, CKM syndrome diagnosis relies on NHANES participant self‐reports, which may lead to a discrepancy from the actual incidence rate. Secondly, despite comprehensive adjustments for covariates in our analytical model, residual confounding factors may still impact the results. Furthermore, the nutritional information originated from self‐documented two 24‐h dietary records, vulnerable to reporting inaccuracies and memory bias. Finally, the observational nature of our study precludes causal inference. Future randomized controlled trials or prospective dietary intervention studies are needed to confirm whether improving DI‐GM scores can directly reduce mortality in CKM syndrome.

## 5 Conclusion

This research reveals that heightened concentrations of DI‐GM among individuals suffering from CKM syndrome correlate strongly with decreased mortality odds, particularly concerning overall and cardiovascular‐related fatalities in patients within CKM stages 0–3, as well as all‐cause mortality rates among those in stage 4.

## Author Contributions

**Shanjiang Chen:** writing – original draft, data curation. **Bin Lin:** data curation, writing – review and editing. **Lianglei Hou:** writing – original draft, data curation, investigation, validation. **Anwu Huang:** writing – original draft, writing – review and editing, formal analysis, data curation, methodology, conceptualization, software. **Qinqin Zhang:** writing – review and editing, supervision, project administration, resources, visualization.

## Funding

This work was supported by the fund project of Wenzhou Science and Technology Bureau (YC20250005).

## Ethics Statement

All NHANES protocols were approved by the NCHS Research Ethics Review Board, and all participants provided written informed consent. As this study utilized publicly available, de‐identified data, it was exempt from additional institutional review board approval.

## Conflicts of Interest

The authors declare no conflicts of interest.

## Supporting information


**Table S1:** Components and scoring criteria of DI‐GM in NHANES.
**Table S2:** Methods for evaluating each CKM stage.
**Table S3:** Multivariable Cox regression analysis of the relationship between DI‐GM and mortality in adults with CKM 0–3 stages.
**Table S4:** Multivariable Cox regression analysis of the relationship between DI‐GM and mortality in adults with CKM 4 stage.
**Table S5:** The number of all‐cause mortality and cardiovascular mortality in each group of DI‐GM.
**Figure S1:** Directed Acyclic Graph (DAG) regarding DI‐GM and all‐cause mortality and cardiovascular mortality.

## Data Availability

All NHANES protocols were approved by the NCHS Research Ethics Review Board, and all participants provided written informed consent. As this study utilized publicly available, de‐identified data, it was exempt from additional institutional review board approval. This study is based on data from the National Health and Nutrition Examination Survey (NHANES) (https://www.cdc.gov/nchs/nhanes/).
