# Supplementary material for: Correlation Between Dietary Index for Gut Microbiota With All‐Cause and Cardiovascular Mortality in Cardiovascular‐Kidney‐Metabolic Syndrome Patients
Source: Food Sci Nutr. 2026 May 28;14(6):e71963. doi: 10.1002/fsn3.71963 (PMC13240121; doi:10.1002/fsn3.71963)
Supplement: Supplementary file 1 — Table S1: Components and scoring criteria of DI‐GM in NHANES. Table S2: Methods for evaluating each CKM stage. Table S3: Multivariable Cox regression analysis of the relationship between DI‐GM and mortality in adults with CKM 0–3 stages. Table S4: Multivariable Cox regression analysis of the relationship between DI‐GM and mortality in adults with CKM 4 stage. Table S5: The number of all‐cause mortality and cardiovascular mortality in each group of DI‐GM. Figure S1: Directed Acyclic Graph (DAG) regarding DI‐GM and all‐cause mortality and cardiovascular mortality. [file FSN3-14-e71963-s001.docx]

**TableS1. Components and scoring criteria of DI-GM in NHANES**

| Components of DI-GM | Included Foods within the Component | Scoring criteria |
| --- | --- | --- |
| Beneficial Gut Microbiota Score | Avocados | For each component, a score of 1 if consumption At or above the sex-specific median, else 0 |
|  | Broccoli |  |
|  | Chickpeas |  |
|  | Coffee |  |
|  | Cranberries |  |
|  | Fermented dairy (including yogurt, cheese,kefir, sour cream, buttermilk) |  |
|  | Fiber |  |
|  | Soybean (including Soymilk,Tofu) |  |
|  | Whole grains (grains defined as whole grains, containing the entire grain kernel—the bran, germ, and endosperm) |  |
|  | Green tea |  |
| Unfavorable Gut Microbiota Score | High-fat diet (% energy) | 0 if consumption at or above 40% energy from fat, else 1 For each remaining component, a score of 0 if consumption at or above the sex-specific median, else 1 |
|  | Processed meat (including frankfurters, sausages, corned beef, and luncheon meat that are made from beef, pork, or poultry) |  |
|  | Red meat (including beef, veal, pork, lamb, and game meat; excludes organ meat and cured meat) |  |
|  | Refined grains (refined grains that do not contain all of the components of the entire grain kernel) |  |
| Abbreviations: DI-GM, Dietary Index for Gut Microbiota; NHANES, National Health and Nutrition Examination Survey. | | |

**TableS2. Methods for evaluating each CKM stage**

| CKM stages | Definition | Criterion | Threshold for CKM conditions |
| --- | --- | --- | --- |
| Stage 0: No  CKM risk factors | Individuals with normal BMI and waist circumference, normoglycemia,  normotension, a normal lipid profile, and no evidence of CKD or subclinical or clinical CVD | All criteria are met | BMI ≥25 kg/m2 (or ≥23 kg/m2 if Asian ancestry)* |
|  |  |  | Waist circumference <88/102 cm in female/male (or if Asian ancestry <80/90 cm in female/male) |
|  |  |  | Participants who do not meet the criteria for other stages |
| Stage 1: Excess or dysfunctional adiposity | Individuals with overweight/obesity,  abdominal obesity, or dysfunctional  adipose tissue, without the presence of other metabolic risk factors or CKD | Any of the three criteria is met | Overweight/obesity |
|  |  |  | Abdominal obesity |
|  |  |  | Prediabetes |
|  |  | All criteria are met | SBP <130 mm Hg and DBP <80 mm Hg without self-reported diagnosis of hypertension or use of antihypertensive medications |
|  |  |  | HDL cholesterol <50/40 mg/dL in female/male and triglycerides <150 mg/dL |
|  |  |  | Low-risk CKD in KDIGO classification according to eGFR and UACR: UACR < 30 mg/g and eGFR ≥ 60 ml/min/1.73m2 . |
|  |  |  | Predicted 10-year CVD risk < 20% |
|  |  |  | No clinical CVD |
| Stage 2:  Metabolic risk  factors and CKD | Individuals with metabolic risk factors (hypertriglyceridemia, hypertension, MetS, diabetes), or CKD | Any of the five criteria is met | Hypertriglyceridemia |
|  |  |  | Hypertension |
|  |  |  | diabetes |
|  |  |  | MetS |
|  |  |  | Moderate-to-high-risk CKD in KDIGO classification |
|  |  | All criteria are met | No very high-risk CKD in KDIGO classification |
|  |  |  | Predicted 10-year CVD risk < 20% |
|  |  |  | No clinical CVD |
| Stage 3:  Subclinical CVD in CKM | Subclinical CVD among individuals with excess/dysfunctional adiposity, other metabolic risk factors, or CKD | Any of the two criteria is met | Very high-risk CKD in KDIGO classification |
|  |  |  | Predicted 10-year CVD risk ≥ 20% |
|  |  | Any of the eight criteria is met | Overweight/obesity |
|  |  |  | Abdominal obesity |
|  |  |  | Prediabetes |
|  |  |  | Hypertriglyceridemia |
|  |  |  | Hypertension |
|  |  |  | diabetes |
|  |  |  | MetS |
|  |  |  | Moderate-to-high-risk CKD in KDIGO classification |
|  |  | The criterion is met | No clinical CVD |
| Stage 4: Clinical CVD in CKM | Clinical CVD among individuals with excess/dysfunctional adiposity,  other metabolic risk factors, or CKD | The criterion is met | Clinical CVD |
|  |  | Any of the nine criteria is met | Overweight/obesity |
|  |  |  | Abdominal obesity |
|  |  |  | Prediabetes |
|  |  |  | Hypertriglyceridemia |
|  |  |  | Hypertension |
|  |  |  | diabetes |
|  |  |  | MetS |
|  |  |  | Moderate-to-high-risk CKD in KDIGO classification |
|  |  |  | Very high-risk CKD in KDIGO classification |

BMI, body mass index; CKD, chronic kidney disease; CKM, cardiovascular-kidney-metabolic; CVD, cardiovascular disease; DBP, diastolic blood pressure; eGFR, estimated glomerular filtration rate; HDL, high-density lipoprotein; KDIGO, The Kidney Disease: Improving Global Outcomes; SBP, systolic blood pressure; UACR, urinary albumin to creatinine ratio.

**TableS3. Multivariable Cox regression analysis of the relationship between DI-GM**

**and mortality in adults with CKM 0-3 stages**

|  | Non-adjusted[HR(95%CI) P] | Adjust I[HR(95%CI) P] | Adjust II[HR(95%CI) P] |
| --- | --- | --- | --- |
| All-cause mortality |  |  |  |
| DI-GM | 0.94 (0.90, 0.98) 0.0033 | 0.89 (0.85, 0.92) <0.0001 | 0.90 (0.87, 0.94) <0.0001 |
| DI-GM group |  |  |  |
| 0-3 | Reference | Reference | Reference |
| 4 | 0.99 (0.79, 1.22) 0.9030 | 1.00 (0.81, 1.25) 0.9702 | 1.02 (0.82, 1.27) 0.8339 |
| 5 | 0.90 (0.73, 1.12) 0.3608 | 0.85 (0.68, 1.05) 0.1294 | 0.88 (0.71, 1.10) 0.2544 |
| ≥6 | 0.82 (0.67, 1.00) 0.0490 | 0.66 (0.54, 0.81) <0.0001 | 0.72 (0.59, 0.88) 0.0017 |
| Cardiovascular mortality |  |  |  |
| DI-GM | 0.89 (0.82, 0.97) 0.0073 | 0.84 (0.77, 0.91) <0.0001 | 0.86 (0.79, 0.93) 0.0004 |
| DI-GM group |  |  |  |
| 0-3 | Reference | Reference | Reference |
| 4 | 1.00 (0.66, 1.50) 0.9882 | 1.02 (0.68, 1.54) 0.9106 | 1.04 (0.69, 1.58) 0.8427 |
| 5 | 0.72 (0.47, 1.12) 0.1470 | 0.68 (0.44, 1.05) 0.0796 | 0.71 (0.46, 1.10) 0.1291 |
| ≥6 | 0.69 (0.47, 1.02) 0.0660 | 0.55 (0.37, 0.82) 0.0032 | 0.61 (0.41, 0.91) 0.0157 |

Non-adjusted model adjust for: None
Adjust I model adjust for: Age;Gender; Race
Adjust II model adjust for: Age;Gender; Race; Marital status;ALT; AST; Urea nitrogen; Total cholesterol; Creatinine; Triglyceride; Uric acid; HDL; LDL; BMI; Diabetes ; Hypertension; Smoke status

**TableS4. Multivariable Cox regression analysis of the relationship between DI-GM**

|  | Non-adjusted[HR(95%CI) P] | Adjust I[HR(95%CI) P] | Adjust II[HR(95%CI) P] |
| --- | --- | --- | --- |
| All-cause mortality |  |  |  |
| DI-GM | 0.94 (0.89, 0.99) 0.0281 | 0.91 (0.86, 0.96) 0.0007 | 0.92 (0.87, 0.97) 0.0043 |
| DI-GM group |  |  |  |
| 0-3 | Reference | Reference | Reference |
| 4 | 0.99 (0.76, 1.29) 0.9390 | 0.98 (0.75, 1.28) 0.8899 | 0.99 (0.76, 1.30) 0.9615 |
| 5 | 0.76 (0.58, 1.01) 0.0597 | 0.71 (0.53, 0.93) 0.0151 | 0.72 (0.54, 0.96) 0.0230 |
| ≥6 | 0.80 (0.63, 1.03) 0.0902 | 0.70 (0.54, 0.90) 0.0052 | 0.73 (0.56, 0.95) 0.0187 |
| Cardiovascular mortality |  |  |  |
| DI-GM | 0.96 (0.88, 1.05) 0.3783 | 0.93 (0.85, 1.01) 0.0972 | 0.93 (0.85, 1.02) 0.1492 |
| DI-GM group |  |  |  |
| 0-3 | Reference | Reference | Reference |
| 4 | 0.95 (0.61, 1.46) 0.8029 | 0.93 (0.60, 1.44) 0.7486 | 0.95 (0.61, 1.49) 0.8310 |
| 5 | 0.66 (0.41, 1.06) 0.0843 | 0.59 (0.37, 0.96) 0.0338 | 0.59 (0.37, 0.96) 0.0342 |
| ≥6 | 0.97 (0.65, 1.44) 0.8634 | 0.83 (0.56, 1.24) 0.3711 | 0.86 (0.57, 1.30) 0.4740 |

**and mortality in adults with CKM 4 stage**

Non-adjusted model adjust for: None
Adjust I model adjust for: Age;Gender; Race
Adjust II model adjust for: Age;Gender; Race; Marital status;ALT; AST; Urea nitrogen; Total cholesterol; Creatinine; Triglyceride; Uric acid; HDL; LDL; BMI; Diabetes ; Hypertension; Smoke status

**TableS5.The number of all-cause mortality and cardiovascular mortality in each group of DI-GM**

| DI-GM | 0 | 1 | 2 | 3 | 4 | 5 | 6 | 7 | 8 | 9 | 10 | 11 | 12 |
| --- | --- | --- | --- | --- | --- | --- | --- | --- | --- | --- | --- | --- | --- |
| All-cause mortality |  |  |  |  |  |  |  |  |  |  |  |  |  |
| 0 | 16  (84.2%) | 157 (86.3%) | 571 (88.8%) | 1526 (90.7%) | 2630 (89.9%) | 2843 (91.4%) | 2236 (91.6%) | 1377 (90.8%) | 688 (93.1%) | 220  (94.0%) | 51  (89.5%) | 10  (100.0%) | 1  (100.0%) |
| 1 | 3  (15.8%) | 25  (13.7%) | 72 (11.2%) | 156  (9.3%) | 296 (10.1%) | 269 (8.6%) | 205  (8.4%) | 139  (9.2%) | 51 (6.9%) | 14  (6.0%) | 6  (10.5%) | 0  (0.0%) | 0  (0.0%) |
| Cardiovascular mortality |  |  |  |  |  |  |  |  |  |  |  |  |  |
| 0 | 18 (94.7%) | 172 (94.5%) | 617 (96.0%) | 1638 (97.4%) | 2834 (96.9%) | 3043 (97.8%) | 2373 (97.2%) | 1474 (97.2%) | 724 (98.0%) | 231 (98.7%) | 56 (98.2%) | 10 (100.0%) | 1  (100.0%) |
| 1 | 1  (5.3%) | 10  (5.5%) | 26 (4.0%) | 44 (2.6%) | 92 (3.1%) | 69 (2.2%) | 68 (2.8%) | 42 (2.8%) | 15 (2.0%) | 3  (1.3%) | 1  (1.8%) | 0  (0.0%) | 0  (0.0%) |


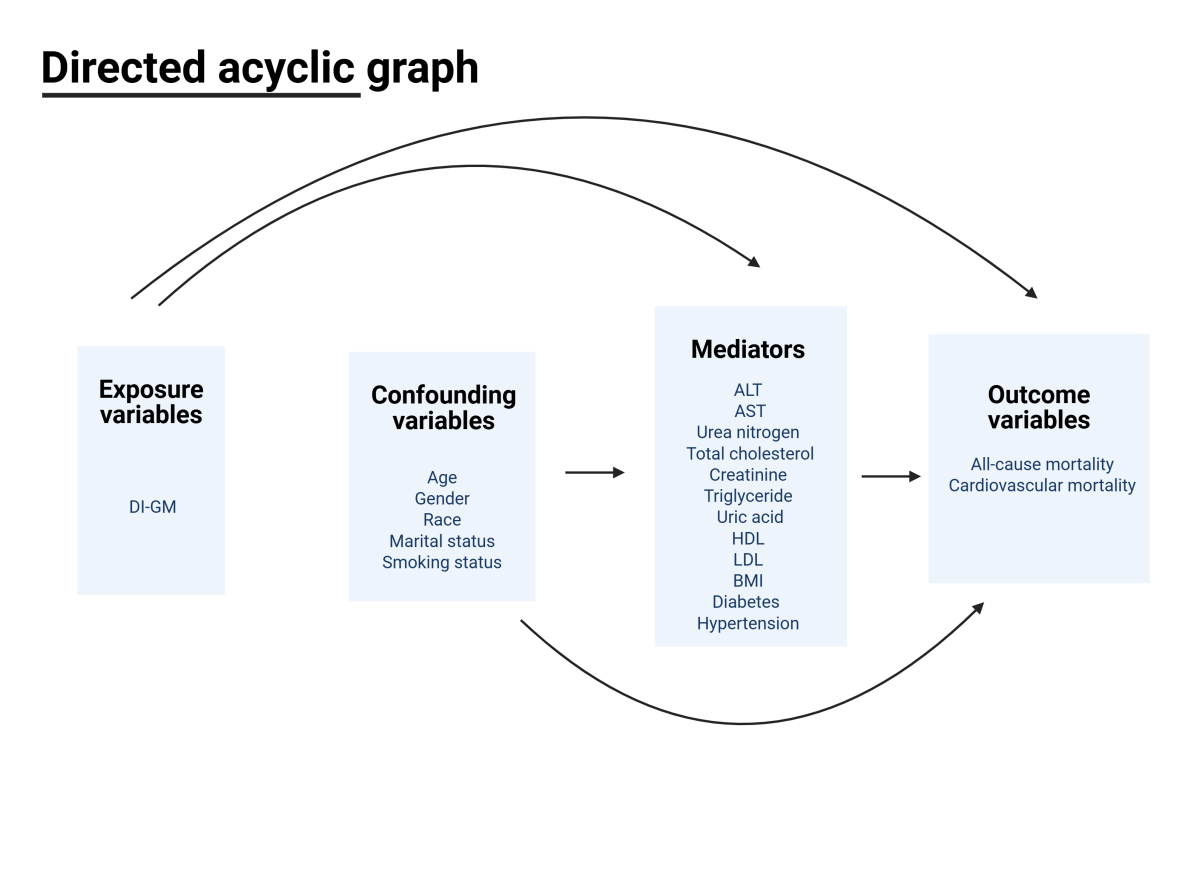


**FigureS1.Directed Acyclic Graph (DAG) regarding DI-GM and all-cause mortality and cardiovascular mortality**
